# Cortical Plasticity as a Mechanism for Storing Bayesian Priors in Sensory Perception

**DOI:** 10.1371/journal.pone.0010497

**Published:** 2010-05-05

**Authors:** Hania Köver, Shaowen Bao

**Affiliations:** Helen Wills Neuroscience Institute, University of California, Berkeley, California, United States of America; University of Maryland, United States of America

## Abstract

Human perception of ambiguous sensory signals is biased by prior experiences. It is not known how such prior information is encoded, retrieved and combined with sensory information by neurons. Previous authors have suggested dynamic encoding mechanisms for prior information, whereby top-down modulation of firing patterns on a trial-by-trial basis creates short-term representations of priors. Although such a mechanism may well account for perceptual bias arising in the short-term, it does not account for the often irreversible and robust changes in perception that result from long-term, developmental experience. Based on the finding that more frequently experienced stimuli gain greater representations in sensory cortices during development, we reasoned that prior information could be stored in the size of cortical sensory representations. For the case of auditory perception, we use a computational model to show that prior information about sound frequency distributions may be stored in the size of primary auditory cortex frequency representations, read-out by elevated baseline activity in all neurons and combined with sensory-evoked activity to generate a percept that conforms to Bayesian integration theory. Our results suggest an alternative neural mechanism for experience-induced long-term perceptual bias in the context of auditory perception. They make the testable prediction that the extent of such perceptual prior bias is modulated by both the degree of cortical reorganization and the magnitude of spontaneous activity in primary auditory cortex. Given that cortical over-representation of frequently experienced stimuli, as well as perceptual bias towards such stimuli is a common phenomenon across sensory modalities, our model may generalize to sensory perception, rather than being specific to auditory perception.

## Introduction

Natural stimuli are variable and often mixed with noise. Our perception of these stimuli is thus derived from ambiguous sensory inputs. Psychophysical experiments in humans and primates indicate that this ambiguity is partly compensated for by incorporating information about the probabilities of previously experienced stimuli directly into the percept in a Bayesian manner [1]–[3]. However, it is not known how this prior information is encoded, retrieved and combined with sensory information by neurons [4], [5].

Previous theoretical investigations of Bayesian inference were often based on homogeneous stimulus representations—i.e., all possible values of stimulus parameters are evenly represented [5]. In such a representational system, prior information is typically modeled as the activation of a sub-population of neurons by top-down influences or cross-modal interactions [5], [6]. This population activity may be linearly combined with sensory-driven activity for optimal integration of information [5]. These prior storage and integration processes are believed to occur in higher-level/multi-sensory cortical areas, but not in low-level sensory cortices.

Although such a mechanism of dynamic prior information encoding and integration may underlie perceptual bias arising in the short-term and in a context-dependent manner [1], it does not account for the often irreversible, robust and context-independent changes in perception that result from long-term, developmental experience [7], [8]. Extensive experience of native speech sounds, for instance, warps the perceptual space so that speech sound variants near a frequently heard prototype are perceived as being more similar to the prototype than they actually are [8], [9]. Such a phenomenon, also known as the perceptual magnet effect, has been interpreted as an example of Bayesian inference in language perception [3], and has been correlated with experience-altered stimulus representations in the sensory cortices [7], [10].

Cortical stimulus representations are not homogeneous. Sensory experience during early development results in robust changes in primary cortical sensory representations that persist into adulthood. A very consistent finding is that more frequently experienced stimuli gain greater representations in primary sensory cortices [7]. The influences of inhomogeneous representations on sensory perception have not been fully explored. We reasoned that the sizes of cortical stimulus representations carry long-term prior information [11], and could play an important role in Bayesian inference in sensory perception. Using a computational model of auditory perception, we investigated the effect of increasing cortical frequency representations on the perception of pure tones. The results indicate that prior information stored in primary auditory cortex frequency representations can be read-out by locally generated neuronal activity and combined with sensory-evoked activity to generate a percept that conforms to Bayesian integration theory.

## Materials and Methods

### Modeling frequency representations in AI

We modeled primary auditory cortex (AI) frequency representations with 800 independent Poisson-firing neurons. The parameters of the model were chosen based on properties of the primary auditory cortical neurons documented in the literature and our unpublished results. In particular, our experimental finding that the firing rates of neurons in auditory cortex exhibit significant variability, with a mean Fano factor value of 0.98+/−0.21 [12], led us to model neuronal firing as a Poisson process. Each neuron had a Gaussian-shaped response-frequency tuning curve as:
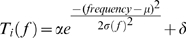
(1)where 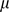 is the characteristic frequency, 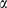 is the maximum response magnitude, 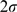 is the tuning bandwidth and 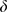 is the baseline spontaneous firing rate. The distributions of tuning bandwidths (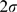) and maximum response magnitudes (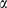) are approximately lognormal, and based directly on our experimental observations. Lognormal distribution is characterized by two parameters—the mean and standard deviation of the logarithm of the investigated response property. The baseline spontaneous firing magnitudes exhibit an exponential distribution, which is characterized by a population mean. The tuning bandwidths, maximum response magnitudes and baseline spontaneous firing magnitudes of the model AI neurons were independently and randomly drawn from the corresponding distributions. The parameters of the distributions are listed in **Table 1**.

**Table 1:** Distribution parameters of neuronal response properties.

Properties	Groups	Mean	SD
Log-bandwidth	Control	−0.7528	0.4727
	7-kHz, BFs of 7 kHz±0.3 octave	−0.8723	0.2837
	7-kHz, other BFs	−0.6359	0.4583
Log-response magnitude	Control	−0.1815	0.5562
	7-kHz	−0.1774	0.5711
Baseline Spontaneous firing magnitude	Control	0.0388	N/A
	7-kHz	0.0374	N/A

**Figure 1:**
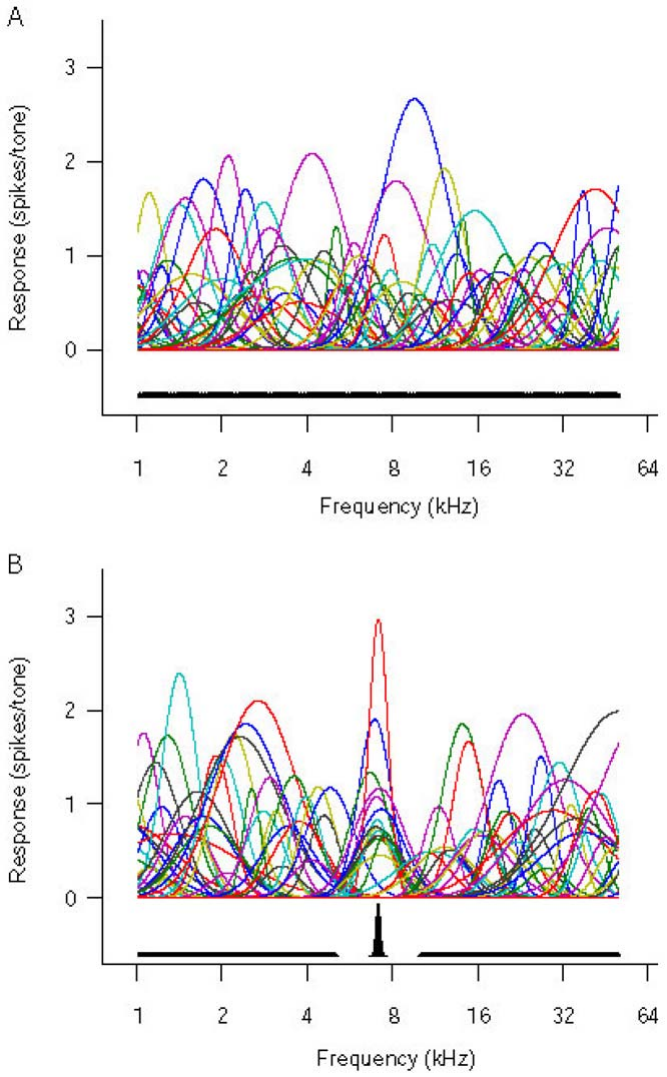
Modeling tonal frequency representations in the primary auditory cortex. (**a**, **b**) Representative tuning curves of the naïve (**a**) and the 7-kHz-over-represented (**b**) model AI. The histograms in the lower part of the graphs show distributions of CFs.

To replicate frequency representations seen in AI of naïve animals and animals with extensive prior experience of a specific tone (7 kHz) [7], model characteristic frequencies (CFs) were either uniformly distributed on a logarithmic scale in the range of 1–32 kHz (naïve) or skewed such that more neurons were tuned to 7kHz (7-kHz-over-represented) (**Fig. 1**). For the 7kHz-over-represented AI, CFs from 5 to 10 kHz were shifted to have a Gaussian distribution centered at 7kHz and with a standard deviation of 0.1 octave (**Fig. 1**). Consistent with our experimental findings the bandwidths of neurons in the over-represented range were slightly smaller **(** **Table 1** **)** [7].

### Modeling frequency perception

We modeled auditory perception by decoding the simulated population response to an input frequency using the maximum-likelihood decoding method [7], [12], [13]. Assume that, when stimulated with a tone of frequency 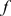, the 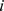
th neuron of the model AI responds with 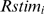 spikes. As the model neurons fire spikes in a Poisson-random fashion, 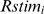 is a Poisson-random number with a mean of 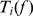, where 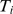 is the neuron's response-frequency tuning curve. The probability of the neuron responding to 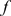 with 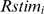 is
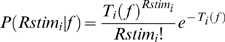
(2)The stimulus likelihood distribution derived from the population response 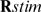 of all N model neurons (1, 2, … N) is:
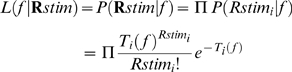
(3)When given the population response to an unknown frequency 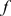, we can calculate the maximum-likelihood estimate of 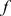, denoted as 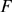, by maximizing the following log-likelihood function [13], [14], using a sequential quadratic programming method [15],
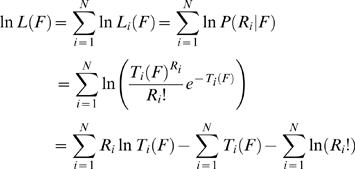
(4)where 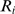 is the response of the 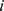
th neuron and in this case refers to 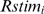 (however, see below).

Modeling Bayesian integration. According to Bayesian integration theory, frequency perception depends both on prior-based expectation and sensory input [1], [3], [16]. In order to return an optimal stimulus estimate, the probability distributions representing each quantity should be combined according to Bayes' rule [1]. The stimulus probability derived from the sensory stimulus-evoked responses 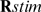 is the frequency likelihood 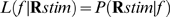. Here we explore the idea that prior probability is read out from the frequency representation by elevated spontaneous activity 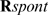 across the whole population of neurons: 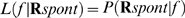. It is important to distinguish 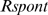 from 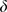, as in contrast to 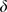, which is part of the neuron's tuning curve and used in the maximum likelihood algorithm, 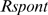 represents elevated spontaneous activity that the maximum likelihood decoder is not aware of.

**Figure 2:**
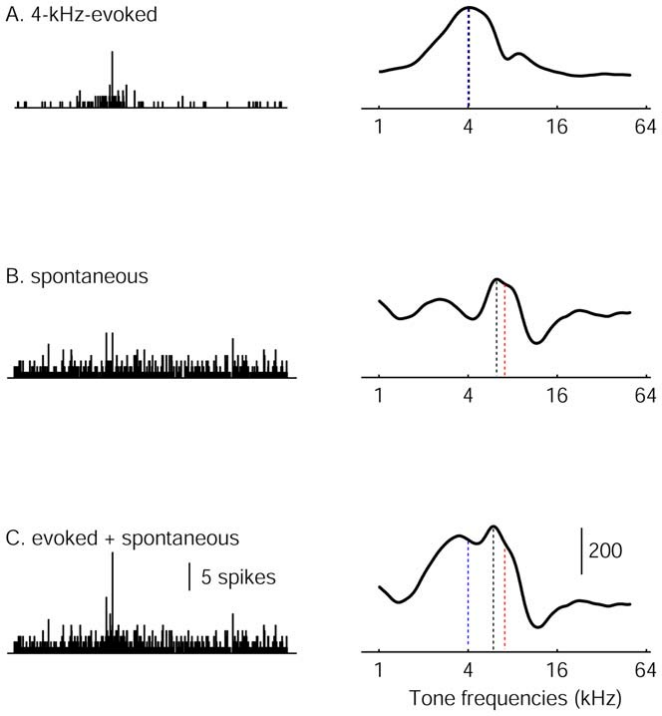
Neuronal population activity and derived log-likelihood functions. Left panels show population activity of the model 7-kHz-over-represented AI, and the right panels show stimulus log-likelihood functions. (**a**) Response of the model to a 4-kHz tone pip (**b**) Elevated baseline activity in the absence of a stimulus (**c**) Summed spontaneous and 4-kHz-evoked activity). Each bar in the left panels represents the firing rate of a model neuron. The neurons are arranged by characteristic frequency, with low frequency-tuned neurons on the left and high frequency-tuned neurons on the right. Blue dotted lines in the right panels show the input frequency, red dotted lines show the over-represented frequency, and the black dotted lines mark the peaks of the log-likelihood functions.

We therefore modeled Bayesian integration of sensory input and prior-based expectation by calculating the stimulus likelihood function derived from the linear superposition of stimulus-evoked activity and elevated spontaneous activity (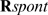 and 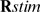)(**Fig. 2c**).
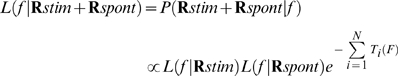
(5)When the frequency representation is homogeneous, equation 5 may be simplified as,
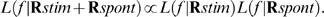
(6)which is in the form of Bayes rule. With inhomogeneous frequency representations, there is a small deviation from Bayes rule caused by an additional term, 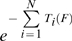 (see equation 5).

## Results

### Convergence of maximum-likelihood estimate at the input stimulus

**Figure 3:**
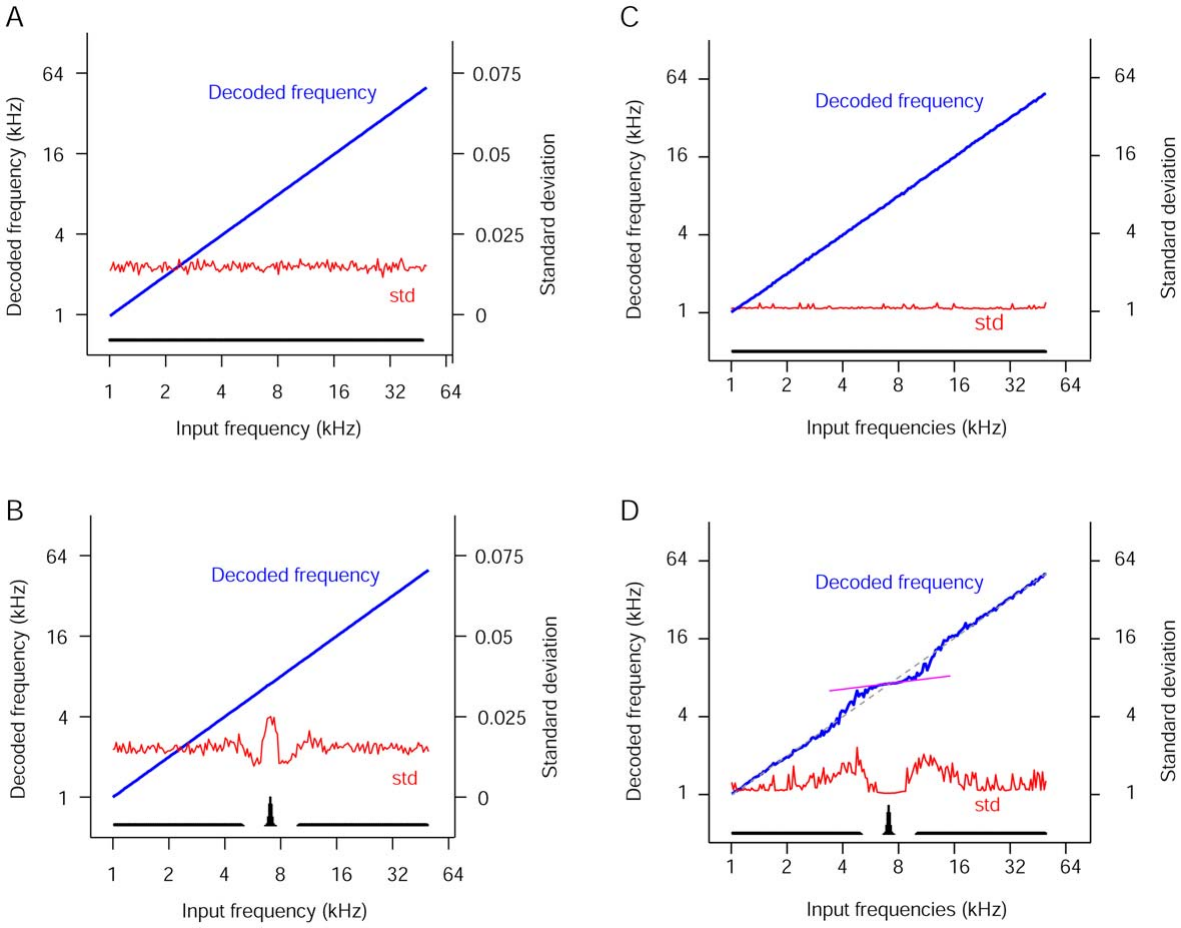
Decoded frequency as a function of input frequency. Both the naïve model AI (**a** and **c**) and 7-kHz-over-represented AI (**b** and **d**) were examined with (**c** and **d**) and without (**a** and **b**) elevated baseline activity. In addition, standard deviation of the decoded frequencies (red) was used to measure the output variability. When baseline activity was elevated in the 7-kHz-over-represented AI, the decoded frequencies show shifts characteristic of Bayesian prior bias (**d**). The pink line shows the slope of the input-output curve at the over-represented frequency. The slope is a measure of the prior bias.

We first examined model auditory perception with normal levels of baseline activity for both the naïve and 7kHz-over-represented model AIs. The maximum likelihood estimate or ‘percept’ converged at the input frequency for both naïve and 7kHz-over-represented model AIs (**Fig. 2a**, **Fig. 3a–b**), even for the under-represented frequencies that no neurons were tuned to. This is not surprising because primary auditory cortical neurons are broadly tuned, and responsive to those frequencies. Thus, the maximum-likelihood estimate of sensory input from population responses is insensitive to inhomogeneity of sensory representations, and always converges on the input stimulus.

### Readout of prior information by nonselectively elevated population activity

We reasoned that the readout of long-term, context-independent priors should not depend on specific patterns of population activity driven by higher-level inferences. Rather, if information about prior stimulus distributions is encoded in the size of primary cortical representations, it should be retrieved by a non-selective increase in the activity in all neurons. Although such activity may be triggered or enhanced by task-related top-down influences or neuromodulatory activity (for example in situations where sensory information is ambiguous) [6], [17], it need not contain specific prior information itself. To test this idea, we increased the baseline activity of all neurons to their maximum response magnitude, and examined the stimulus likelihood distribution in the absence of stimulus-evoked activity (**Fig. 2b**). The likelihood function of the naïve model AI was flat with no peaks (data not shown), whereas that of the 7kHz-over-represented model AI showed a peak near the over-represented frequency (**Fig. 2b**). This peaked likelihood function may be regarded as an internal representation of the prior probability distribution of the stimulus. In calculating the likelihood function here, we assumed that the maximum-likelihood decoder was unaware that the elevated activity was not sensory driven. This is not different from the treatment of top-down prior-related or cross-modal activity in other models of Bayesian inference [5] (see Discussion).

### Bayesian Integration of prior and sensory information

It has recently been shown that Bayesian integration of probability distributions represented in neuronal population codes such as the one used in our model may be achieved by simple summation of population activities [5]. Stimulus-evoked and spontaneous activity in primary sensory cortices summates linearly [18]. When we decoded the summed population response (consisting of the linear superposition of elevated baseline activity and 4-kHz-evoked activity [5]), the peak of the likelihood function was shifted towards 7kHz for the 7kHz-over-represented model (**Fig. 2c**, right). Such a shift was observed for frequencies near 7kHz in the 7-kHz-overrepresented (**Fig. 3d**), but not the naïve (**Fig. 3c**), model AI. This perceptual bias is consistent with Bayesian integration of prior information and noisy auditory input [3], and may explain the impaired discrimination ability for frequencies near over-represented frequencies which has been recently reported [7].

### Decoding variability

The relative decoding variability at the over-represented frequency range behaves differently with and without the elevated baseline activity. With an increased baseline, although overall variability is increased, it is relatively lower for the over-represented frequencies than for the neighboring frequencies (**Fig. 3d**). This is consistent with human psychophysical studies showing that extensively experienced native speech sounds are perceived with less variability than novel foreign speech sounds [19].

### Influences of neuronal population size and activity levels on perceptual bias

**Figure 4:**
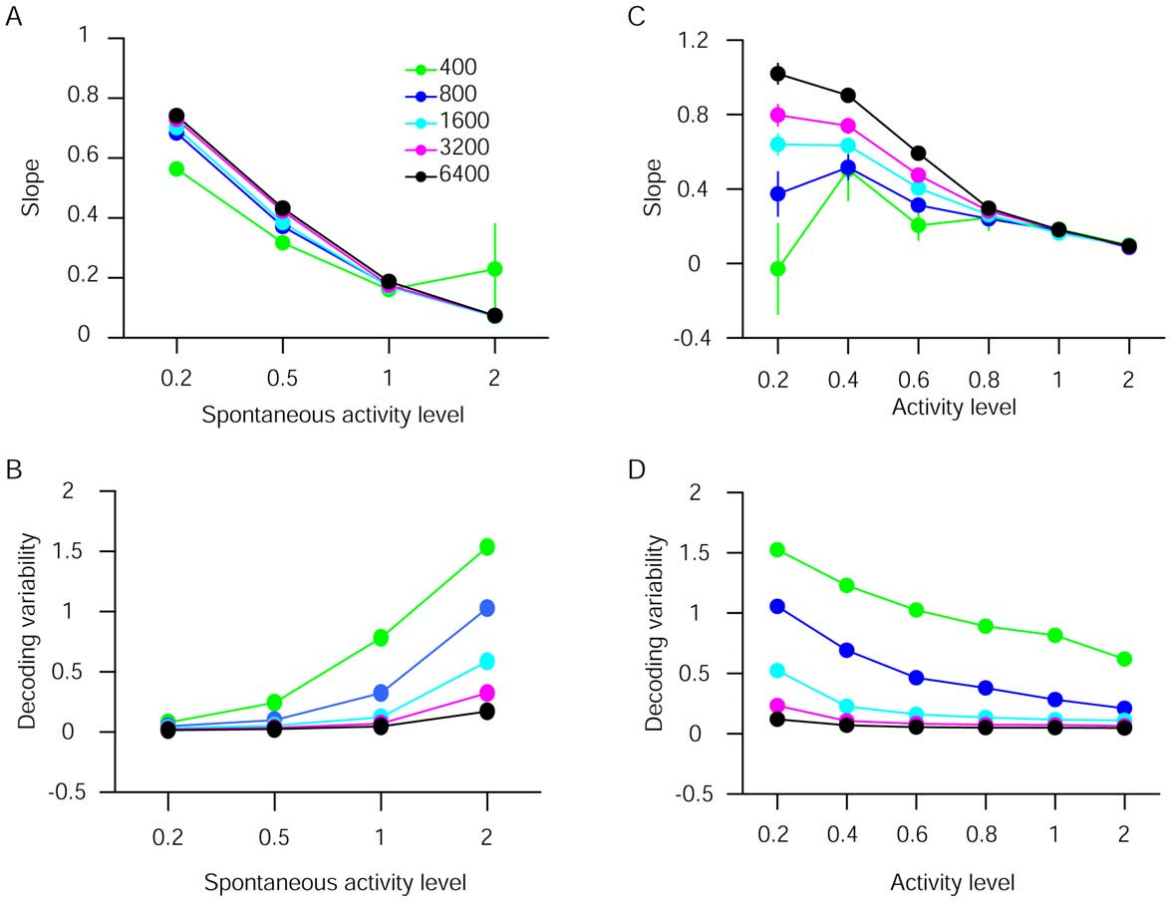
Influence of neuronal population size, baseline activity level, and overall activity level on sensory decoding. (**a** and **c**) Slopes of the input-output function (see the pink line in Figure 3D), showing the degree of prior bias. (**b** and **d**) Standard deviation of the decoded frequencies, which measures the decoding variability. In **a** and **b**, sensory-evoked activity level was fixed and the neuronal population size (color-coded) and baseline activity level were systematically varied. Baseline activity level refers to the multiplicative factor. For example, baseline activity level of 2 indicates doubling of activity. In **c** and **d**, the ratio of baseline activity to maximum evoked response magnitude was set at 1, and activity was systematically varied together. Error bars represent SEM, and are mostly masked by the data symbols.

Some parameters of the model AI, such as the total number of neurons and the magnitude of the elevated spontaneous firing rate, were arbitrarily chosen. We therefore systematically varied these parameters to explore their influence on the observed characteristic perceptual shift (**Fig. 4**). The slope of the input-output function in the over-represented frequency range was used as a measure of perceptual shift magnitude—smaller slopes indicate greater prior bias (**Fig. 3d**). When the magnitudes of the stimulus-evoked responses were fixed, increasing the level of baseline activity led to smaller input-output slopes, indicative of stronger prior biases (**Fig. 4a**). Similarly, when the ratio of baseline to evoked responses was set at 1, increasing overall activity also resulted in stronger prior biases (**Fig. 4c**). Increasing baseline activity led to higher decoding variability (**Fig. 4b**), whereas increasing both baseline and sensory-evoked activity reduced decoding variability (**Fig. 4d**). Increasing neuronal population size reduced this variability. Thus, higher baseline-to-evoked activity ratio in a larger population of neurons would produce more reliable and robust prior biases. Optimal integration of prior and sensory information may be achieved by adjusting the levels of baseline activity in a task-dependent manner (e.g., higher baseline activity when the stimulus is more ambiguous).

## Discussion

Earlier studies have suggested that dynamic prior information may be encoded by the activity of a subset of primary cortical neurons in a homogeneous representational system. The specific pattern of activity is driven by inputs outside of primary sensory cortex that carry prior information derived from high-level inference. Thus the encoding of the prior is separate from its integration with sensory information and must be mediated by different neural circuits. The specific brain substrates and mechanisms for prior encoding and retrieval are unknown. The present study considered the possibility of storing long-term prior information in the size of sensory representations. A novel finding is that in the context of auditory perception, long-term priors about sound frequency distributions can be retrieved by *non-selective* increase in the activity of *all* neurons in primary auditory cortex. In the model, the same cortical circuit performs both the encoding and integration of the prior. The increase in overall activity could be driven by a general top-down signal without specific prior information.

In order for optimal Bayesian integration of prior and sensory information to occur, our model requires that the relative contributions of prior-related and sensory-evoked activity be modulated by task conditions on a trial-by-trial basis. In other words, although the prior is long-term, optimal Bayesian inference requires that the extent to which it used in generating a sensory percept depend on task demand and stimulus uncertainty. Our simulation shows that this could be accomplished by changing overall levels of activity. Higher levels of overall activity increase the contribution of prior information to sensory perception and increase prior bias. Thus our results suggest that in situations where auditory input is ambiguous, the overall level of activity in all primary auditory cortex neurons should increase. Although dynamic prior encoding also calls for a higher level of prior-related activity when the sensory input is ambiguous, such activity occurs only in a subset of neurons.

Elevated neuronal activity is not the only way that a prior stored in the size of sensory representations could be read out. Another possibility, recently proposed in unpublished work [20], is that the decoder is unaware of the change in sensory representations: such a scheme leads to the same degree of prior bias as our simulation. The major difference between these two schemes is that in our simulation the degree of bias is adjustable and dependent on task conditions rather than being a fixed and inbuilt property of the decoder. Recent experimental work has suggested that the degree of bias for long-term priors may be dependent on task conditions [21].

Different levels of intrinsic, “baseline” activity in primary sensory cortices have been shown to profoundly influence neuronal responses to sensory stimuli [18], [22], sensory perception [23] and motor behaviors [24]. In our model, the level of internally driven activity depends on the uncertainty of auditory input. It remains to be determined how this sensory uncertainty is encoded and used to optimize performance. Task-related uncertainty has been shown to modulate baseline activity [6], possibly by activation of neuromodulatory systems, thereby influencing the extent to which behavioral responses depend on internal prior information versus external sensory information sources [17]. Another possibility is that the background noise that characterizes ambiguous sensory situations nonspecifically activates auditory cortex to achieve the same end as elevated spontaneous activity. However, unlike elevated spontaneous activity, noise activates neurons in different regions of auditory cortex differentially [25] and its effects can therefore not be directly inferred from this study.

Maximum-likelihood estimation is an unbiased feature decoding method. With a sufficient number of neurons, as well as the knowledge of which part of the neuronal activity is due to the input stimulus, its decoding result always converges on the input stimulus (**Fig. 3**). In earlier studies of Bayesian integration, top-down prior-related activity and cross-modal sensory activity were linearly combined with, and not distinguished from, stimulus driven activity [5]. Perceptual biases arise out of this treatment of prior-encoding or cross–modal activity. We treated spontaneous activity similarly in our simulation – the decoder does not distinguish it from stimulus driven activity.

Elevating spontaneous activity results in greater decoding variability in our simulations (**Fig. 3** **)**. Thus, stimulus-decoding performance is decreased. However, the increase in spontaneous activity in our model is caused by task demand when the sensory input is ambiguous, and cannot be resolved by simple (optimal) stimulus decoding. It enables integration of prior information to optimally resolve stimulus ambiguity. Furthermore, decoding variability decreases rapidly when more neurons are included in the model (**Fig. 4**), and therefore may not pose a problem for the real brain.

Although our model is based on tonal frequency representations in primary auditory cortex, it should generalize to any stimulus dimension represented by populations of plastic sensory neurons. Over-representation of frequently experienced stimuli is a common feature of primary sensory cortex independent of modality, and occurs for sound intensity [26], sweep direction [27], spectral bandwidth [25] and temporal rate [28] in primary auditory cortex, line orientation [29] in primary visual cortex, and whisker representation in primary somatosensory cortex [30], to name a few examples. Maximum likelihood estimation has also been used to model sensory perception in multiple modalities [13], [14]. Although there are not many explicitly documented examples of perceptual bias towards long-term priors outside of the auditory system, recent work in the visual system has shown that subjects perform a line orientation discrimination task in a way that suggests bias towards line orientations that occur more frequently in the environment [31]. Our model may therefore generalize to sensory perception in general, rather than the specific case of auditory perception.

In summary, we have shown that long-term prior information in auditory perception may be stored in the sizes of primary auditory cortex frequency representations and be read out by non-selective increases in baseline activity. Such increase in baseline activity may be controlled by task demand through top-down influences, and when combined with stimulus-driven activity, allow Bayesian integration of prior and sensory information. Our model makes two unique testable predictions independent of sensory modality that distinguish it from other models of dynamic Bayesian integration: 1) percepts of ambiguous stimuli are biased toward stimuli with larger sensory representations; 2) ambiguous sensory input leads to a *non-selective* increase in baseline activity of all coding neurons.
